# The Role of MicroRNAs in Regulating Cytokines and Growth Factors in Coronary Artery Disease: The Ins and Outs

**DOI:** 10.1155/2020/5193036

**Published:** 2020-07-25

**Authors:** Armita Mahdavi Gorabi, Nasim Kiaie, Thozhukat Sathyapalan, Khalid Al-Rasadi, Tannaz Jamialahmadi, Amirhossein Sahebkar

**Affiliations:** ^1^Research Center for Advanced Technologies in Cardiovascular Medicine, Tehran Heart Center, Tehran University of Medical Sciences, Tehran, Iran; ^2^Department of Academic Diabetes, Endocrinology and Metabolism, Hull York Medical School, University of Hull, Hull, UK; ^3^Medical Research Centre, Sultan Qaboos University, Muscat, Oman; ^4^Biotechnology Research Center, Pharmaceutical Technology Institute, Mashhad University of Medical Sciences, Mashhad, Iran; ^5^Department of Food Science and Technology, Quchan Branch, Islamic Azad University, Quchan, Iran; ^6^Department of Nutrition, Faculty of Medicine, Mashhad University of Medical Sciences, Mashhad, Iran; ^7^Halal Research Center of IRI, FDA, Tehran, Iran; ^8^Biotechnology Research Center, Pharmaceutical Technology Institute, Mashhad University of Medical Sciences, Mashhad 9177948564, Iran; ^9^Neurogenic Inflammation Research Center, Mashhad University of Medical Sciences, Mashhad, Iran; ^10^School of Pharmacy, Mashhad University of Medical Sciences, Mashhad, Iran

## Abstract

Coronary artery diseases (CAD), as a leading cause of mortality around the world, has attracted the researchers' attention for years to find out its underlying mechanisms and causes. Among the various key players in the pathogenesis of CAD cytokines, microRNAs (miRNAs) are crucial. In this study, besides providing a comprehensive overview of the involvement of cytokines, growth factors, and miRNAs in CAD, the interplay between miRNA with cytokine or growth factors during the development of CAD is discussed.

## 1. Introduction

Coronary artery disease (CAD) involves all diseases in which blood flow to the heart muscles is restricted. Plaque formation resulting from coronary atherosclerosis is the main reason for blood flow restriction. Destabilization and subsequent rupture of plaque might produce acute coronary syndrome (ACS), which is classified into unstable angina (UA), ST-segment elevation myocardial infarction (STEMI), and acute myocardial infarction (AMI) which differ in the extent of involvement of cardiac muscles and release of cardiac markers [1].

Cytokines, a broad category of small polypeptides of less than 80 kDa including chemokines and growth factors that are released from cells locally and potentially contribute to the initiation of coronary artery disease [2]. Inflammatory cytokines and growth factors are involved in various pathways involved in the development of CAD including STAT, MAPK, and SMAD [3, 4].

miRNAs are a class of small (20-25 nucleotides) noncoding RNAs which affects various molecular pathways, altering gene expression and regulating cytokine production [5]. The mechanism by which miRNA regulates gene expression includes miRNAs binding to the 3′ untranslated region (UTR) of the target gene, destabilizing the mRNA, translation repression thereby inhibiting protein synthesis [6]. To protect miRNA from degradation by plasma enzymes, they are packed in exosomes, proteins, and most abundantly in CD31+ microparticles, which are released from the endothelial cells (ECs) and platelets [7–9]. Besides this packaging mechanism, miRNAs which are highly stable in plasma makes it a suitable choice for use as diagnostic biomarkers for various disorders [10].

Both cytokines and miRNAs are components of the signaling pathways that carry out essential cellular functions, and in some cases, drive disease progression. In this review, the distinct contribution of cytokines, growth factors, and miRNAs in CAD is presented, and then the relationship between miRNA and cytokine or growth factors in CAD progression is reviewed.

## 2. CAD-Related Cytokines and Growth Factor

It is well established that inflammation is an integral part of CAD. Therefore, inflammatory cytokines in the peripheral blood produced after caspase-1 pathway activation from macrophages and lymphocytes, are important players in the progression of CAD [11]. Serum levels of T helper cytokines, including proinflammatory Th1 type and anti-inflammatory Th2 type cytokines, are good CAD risk indicators. In general, stimulation of Th1 and Th2 suppression contributes to the development of CAD following type 2 diabetes mellitus [12]. Th1 type, especially interferon-gamma (IFN-*γ*), has a prominent role in the initiation of CAD and transforming stable angina to unstable angina via macrophage activation, weakening of the atherosclerotic plaque's fibrous cap and plaque rupture [13, 14]. In contrast, Th2 cytokines such as interleukin IL-4 and IL-10 deactivate macrophages and stabilize the plaque [15].

Circulating levels of IL-1*β* and its receptor are a significant marker of atherogenesis and CAD [11, 16]. IL-18 is another cytokine that is related to the severity of CAD [17]. In one clinical study, patients with ACS had not only higher IL-1*β* and IL-18 but also IL-6, a downstream cytokine to the two cytokines above than in patients with stable CAD [18]. However, the role of IL-6 in CAD is complex since elevated plasma levels of IL-6 are associated with cardiovascular risk and formation of atherosclerotic plaque, it could exert an inhibitory effect on other inflammatory cytokines such as IL-1 [19]. An in vitro study on the peripheral blood mononuclear cells of patients with CAD showed that expression of cytokines including CCL2, CXCL8, CXCL9, CXCL10, IFN- *γ*, and IL-10 increase in both stable or unstable angina (UA) groups. The only difference between these two groups was lower IL-10 mRNA expression in the UA group [20]. The ratio of IL-18/IL-10, as well as levels of C-reactive protein (CRP), a cytokine mediator, is considered diagnostic tools for premature CAD with high sensitivity and specificity. An elevated CRP represents more severe CAD [21].

Bolez et al. demonstrated that increased plasma level of INF-*γ*, tumor necrosis factor-alpha (TNF-*α*), IL-1*β*, and IL-8 cytokines, as well as reduced secretion of IL-2 from activated T-cells and IL-4 from T helper cells (Th2 type), is a characteristic of coronary artery ectasia, a kind of CAD distinguished by at least 1.5-fold expansion of the artery [22]. TNF-*α* with genetically altered promoter region affects the development of CAD in patients suffering from nonalcoholic fatty liver disease. Patients carrying TNF-*α*-238 guanine to alanine (GA) polymorphism are more prone to the development of CAD [23].

A study of 180 patients with stable angina showed NOGO-B/NUS1 and TL1A/DR3 cytokines were increased in CAD. The ADAMTS-5 cytokine, which is expressed in the macrophages during the differentiation of monocytes to macrophages, were increased in CAD while it was reduced in patients with peripheral artery disease (PAD). TNF-like cytokine 1A (TL1A) axis, after interaction with its receptor, death receptor 3 (DR3), initiates proinflammatory pathways in atherosclerosis [24].

Clinical studies also showed there is a direct correlation between increased levels of proinflammatory cytokines (IFN-*γ*, TNF*α*, IL-2, IL-6, IL-9, and IL-17) and anti-inflammatory cytokines (IL-4 and IL10) with the severity of CAD determined by coronary angiography [25, 26]. In a retrospective case-control study, IL-5 level was associated with coronary heart disease. The cytokine, which was most strongly associated with the risk of coronary heart disease, was IL-6 [27]. Increased number of Th9 cells and its related cytokine, IL-9, were also associated with atherosclerosis through mediating infiltration of inflammatory cells into the atherosclerotic plaques [28]. Suppression of transforming growth factor-beta (TGF-*β*) cytokine is another characteristic feature of CAD [29]. A meta-analysis of prospective studies showed that there was no significant correlation between heart disease and the levels of soluble forms of CD40 ligand (sCD40L) and matrix metalloproteinase (MMP)-9 cytokines [30].

## 3. CAD-Related miRNAs

Numerous studies have shown that miRNAs are involved in CAD and related conditions owing to the role of miRNAs in regulating the function of cells, including endothelial cells (EC), smooth muscle cells (SMC), and macrophages, as well as in inflammation and other metabolic processes. The expression of miRNAs is specific to the cell type. For example, miRNA-222 is expressed in endothelial cells and vascular SMCs, 126-3p, and miR-21 are detected in ECs of vascular tissues, platelets, and bone marrow-derived cells and miR-499 and miR-133a are expressed in muscle cells [31–33]. In CAD and related diseases, expression of these miRNAs is up or downregulated. An example is the upregulation of fibrosis-related miRNAs (miR-29b) and inflammation-related miRNAs (miR-124a, miR-146a, miR-155, and miR-223) in abdominal aortic aneurysm (AAA) [34]. After upregulation, some miRNAs are released into the circulation. For example, miR-499 and miR-133a are released into the blood following myocardial injury [32, 33]. The circulating levels of miRNAs, as illustrated in Table 1, increase or decrease in response to various coronary diseases. These changes in the level of miRNAs in extracellular fluids and circulation open new doors for the early detection of CAD. A number of these miRNAs and their alteration in response to disease are evaluated clinically for diagnostic purposes.

Among these miRNAs, some have more sensitivity and specificity to the disease model. For example, increased levels of miR-21, miR-92, miR-126, and miR-132 are highly specific to the UA [35–40], while a combination of miR-126, miR-197, and miR-223 is specific for AMI [41]. There are confounding factors in the measurement of miRNA levels that should not be forgotten. Studies have shown that many factors, including administration of heparin, aspirin, antiplatelet agents, statins, and angiotensin-converting enzyme (ACE) inhibitors before blood sampling from patients, as well as endogenous heparin can affect miRNA quantification [42–47].

Measuring the circulating levels of some miRNAs enables the assessment of disease severity. For example, expression of miR-574-5p in CAD increases proportionally to the severity of the disease [48]. Furthermore, coronary artery calcification leads to reduced expression of miR-138-2-3p, miR-1181, miR-6816-3p, and miR-8059, among them the correlation between miRNA-8059 expression and the level of coronary calcification predicts the severity of the disease [49].

Some miRNAs, such as miR-126-3p, miR-132, miR-140-3p, miR-197, miR-210, and miR-223 are not only involved in the various coronary diseases but also could predict the mortality in angiographically documented CAD patients [50].

Some miRNAs are correlated to the composition of atherosclerotic plaques. For example, miR-100 is released from vulnerable coronary plaques, and its plasma levels are associated with plaque composition as determined by integrated backscatter intravascular ultrasound (IB-IVUS). miR-100 is strongly correlated with the percentage of lipid volume and negatively correlated with fibrous volume [51].

## 4. Regulating CAD-Related miRNAs by Cytokines and Growth Factors

**Figure 1:**
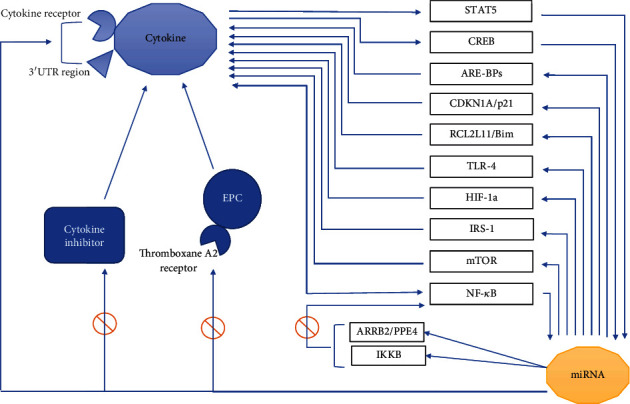
The interplay between miRNAs and cytokines involved in CAD. Cytokines can alter the expression of miRNAs through the induction of miRNA transcription factors such as CREB or activating signaling pathways such as STAT5 and NF-*κ*B. Conversely, miRNAs change the production, stability, and release of cytokine via direct targeting of 3′UTR region of cytokine or cytokine receptors, affecting ARE-BPs, CDKN1A/p21, BCL2L11/Bim, IRS-1, and mTOR mediators, and activating or blocking signaling pathways such as IKK*β*/NF-*κ*B, IRRB2/PPE4/NF-*κ*B, TLR4, and HIF-1a. An indirect effect of miRNA on cytokines is mediated by blocking thromboxane A2 receptors of EPCs which inhibits cytokine signaling. CREB: cAMP-response element-binding protein; ARE-BPs: binding proteins containing adenine and uridine elements; TLR4: toll-like receptor 4; IKK*β*: I*κ*B kinase *β*; IRS-1: insulin receptor substrate 1; mTOR: mammalian target of rapamycin.

While many studies dealt with the role of miRNAs or cytokines in CAD, it should be noticed that the contribution of both miRNA and cytokine in CAD is more complicated since these two factors affect each other's expression (see Figure 1). Researchers have been trying to elucidate the mechanism by which miRNAs affects CAD and related disease processes by studying the most probable target genes for CAD-related miRNAs using targetscore algorithm [52]. Paying attention to the relationship between miRNA, cytokines, and growth factors not only shed light on these mechanisms but also leads to a better understanding of whether miRNA or cytokine and growth factors are more suitable targets for CAD therapeutics.

### 4.1. Growth Factors

Growth factors are important cytokines that could alter miRNA expression. Treatment of ECs with vascular endothelial growth factor (VEGF) results in the induction of miR-17–92 cluster and alteration of miRNA expression [53]. Treatment of human umbilical vein endothelial cells (HUVECs) with VEGF and beta fibroblast growth factor (bFGF) upregulated miR-132 3–6 h after treatment via induction of cAMP-response element-binding protein (CREB), an essential element for miR-132 transcription [54]. Suarez et al. showed that treatment of HUVECs with proinflammatory cytokine, TNF, increased the expression of miR-17-5p, miR-31, miR-155, and miR-191 [55]. Exposure of ECs to IL-3 or bFGF, activators of the STAT5 signaling pathway, decreased the expression of miR-222 [56].

### 4.2. Inflammatory Cytokines

A correlation exists between miRNAs and proinflammatory cytokines so that signal intensity of monocyte chemoattractant protein (MCP-1) cytokine decreased with increasing expression of miR-22, miR-124, miR-146a, and miR-223 [34, 57]. Likewise, such relation exists between TNF-*α* and miR-126 and miR-19b, or TGF-*β* and miR-146a [34, 58]. Additionally, IL-1*β* induce miR-146a/b expression in ECs [59]. THP-1 cells, a monocytic cell line, when treated with IL-1*β*, activated TLR/IL-1R signaling which leads to NF-*κ*B activation and increased miR-146a expression up to 15-fold during 24 h [60]. Stimulating miR-29 mimics-transfected immortalized human bronchial epithelial cells (BEAS-2B) as a model of allergic inflammation with TNF-*α* and IL-4 cytokines for 48 hours lead to both upregulation of endogenous miR-29 expression and increasing soluble ST2, an inflammation-related gene and a receptor for IL-33. However, miR-29 overexpression decreased soluble ST2 mRNA expression [61].

## 5. Regulating CAD-Related Cytokines and Growth Factors by miRNAs

miRNAs regulate cytokine and growth factors production and release by two distinct mechanisms: they directly bind to 3′UTR target site in cytokine, or they affect binding proteins containing adenine and uridine elements (ARE-BPs) and consequently affect cytokine or growth factors stability and production. Some ARE-BPs include tristetraprolin (TTP), AU-rich binding factor 1 (AUF1) and members of the Hu protein R (HUR) family, which exist in some, but not all cytokines [62].

### 5.1. VEGF

miRNAs might directly target growth factors. CAD-related miRNAs including *miR-16*, *miR-20a*, *miR-20b*, *let-7b*, *miR-17-5p*, *miR-27a*, *miR-106a*, *miR-106b*, *miR-107*, *miR-193a*, *miR-210*, *miR-320*, and miR-361, have been recognized to target VEGF and bind VEGF 3′UTR through nt160–195 binding site [63]. Among CAD-related miRNAs, miR-15a, miR-16, miR-93, miR-200b, miR-361-5p, and miR-424 repress VEGF expression by affecting VEGF receptors, while miR-23, miR-126, miR-132, and miR-221 negatively regulate VEGF signaling pathway by targeting downstream of VEGF [64, 65]. In addition, miR-23a secreted from endothelial progenitor cells (EPCs) of CAD patients targets epidermal growth factor receptor (EGFR) and suppresses VEGF [66]. miRNA-31 and miRNA-720 block FAT4 and thromboxane A2 receptors of EPCs in CAD patients [67]. Since thromboxane A2 receptors have an inhibitory effect on VEGF signaling [68], miRNA-31 and miRNA-720 indirectly increase VEGF-induced angiogenesis. Fish et al. found that the response of ECs to VEGF is regulated by miR-126. The beneficial effect of miR-126 on this cytokine is the inhibition of Sprouty-related protein SPRED1 and phosphoinositol-3 kinase regulatory subunit 2 (PIK3R2/p85-beta), two inhibitors of VEGF pathway, and therefore preserving VEGF signaling and maintaining vascular integrity [69]. In other disease models, such as tumor angiogenesis, the elevation of VEGF expression and secretion is observed following transfection of miR-181a in chondrosarcoma cell line JJ [70]. It worth to notice that the levels of miR-181a reduction in ACS is not in favor of angiogenesis. miR-103 also decreased VEGF expression and vascular density in rats which underwent middle cerebral artery occlusion [71]. Transfecting SK-N-AS cell line with miR-93-5p altered expression of VEGF and IL-8, showing that the 3′UTR region of these cytokines is a target for miR-93-5p [72].

### 5.2. TGF-*β*

TGF-*β* signaling, a critical factor for putting the stable atherosclerotic plaques at the risk of rupture, is affected by CAD-related miRNAs such as miR-21, miR-25, and miR-106b via the mechanism of affecting CDKN1A/p21 and BCL2L11/Bim [73–75]. miR-21 targets TGF- *β* and bone morphogenic protein (BMP) which results in the induction of contractility in vascular smooth muscle cells [76, 77]. Increasing miR-133a expression suppresses TGF-*β*1 and connective tissue growth factor (CTGF) expression. CTGF is an important cytokine produced from fibroblasts and downstream for TGF-*β*1 profibrotic pathway [78]. Therefore, miR-133a exerts a cardioprotective effect via suppressing this pathway and reducing fibrosis in MI [79].

### 5.3. Interleukins

Some CAD-related miRNAs, including miR-21, miR-126, miR-146a, miR-155, and miR-223 are considered to be inflammatory-related miRNAs [80]. Therefore, it is highly probable that these miRNAs affect the inflammatory cytokines to pull the inflammatory processes together. During atherosclerosis, apoptotic bodies are released from ECs which contain miRNAs such as miR-126. The release of these apoptotic bodies (or miR-126) stimulates CXCL12 production [81]. miR-146a/b affects toll-like receptor 4 (TLR4) signaling through regulating IL-1 receptor-associated kinase 1 (IRAK1) and TNF receptor-associated factor 6 (TRAF6), two downstream molecules to TLR4 [60, 82]. TLR4 stimulation by miRNAs is necessary since activation of this protein initiates signaling cascades, which lead to proinflammatory cytokine production [83] which is the reason behind the regulatory role of miR-146a in inflammation during CAD progression [84]. Transfecting human vascular endothelial cell line (EA.hy926 cells) with miR-146 mimic showed that this miRNA contributes to the inflammatory processes in sepsis disease via decreasing expression of inflammatory cytokines, including TNF-*α*, IL-6, and intercellular adhesion molecule (ICAM)-1, and E-selectin [85]. Transfecting natural killer cells with lentiviral vectors expressing miR-155 decreased the pro-inflammatory cytokines such as IL-1, IL-6, TNF-*α*, and IFN-*γ* [86]. A positive correlation also exists between IL-17 production by CD4+ T cells and miR-155 expression [87].

A study on human nasal epithelial cells (JME/CF15) treated with IL-13 as an in vitro model of allergic rhinitis showed there is association between miR-16 and inflammatory reactions so that miR-16 upregulation in JME/CF15 cells leads to inhibition of cytokines including granulocyte-macrophage colony-stimulating factor (GM-CSF), eotaxin, IL-1*β*, IL-6, and IL-10. The mechanism of the effect of miR-16 on inflammatory cytokines is governed by blocking IKK*β*/NF-*κ*B signaling pathways because miR-16 inhibit either I*κ*B kinase *β* (IKK*β*) expression or NF-*κ*B activation [88]. Intranasal administration of an inhibitor of miR-21 (anti-miR-21 antagomir) to ovalbumin sensitized BALB/c mice, as a model of acute bronchial asthma, reduced the levels of IL-4 and airway inflammation [89]. A recent study found that transfecting CD4 T cells with miR-31 reduce the expression levels of IL-2 and IL-4 via targeting NF-*κ*B and HIF-1*α* [90]. miR-150 is a negative regulator of IL-2 due to targeting ARRB2, repressing ARRB2/PDE4 and inhibiting NF-*κ*B pathway [91].

### 5.4. IFN-*γ*

miR-21 transfected w.t. B6 T cell showed upregulation of proinflammatory cytokines including TNF-*α* and IFN-*γ* [92]. IFN-*γ* expression is also negatively regulated by miR-24 and miR-29 due to the presence of a target site for these miRNA on IFN-*γ*-3′UTR [93, 94]. Systemic administration of miR-17 or miR-19b also produces inhibition of IFN-*γ* [95]. In addition, expression of IFN-*γ* in CD4^+^ T cells increase after knockdown of miR-126 via the mechanism of enhancing the expression of insulin receptor substrate 1 (IRS-1) as a target for miR-126 [96].

### 5.5. TNF-*α*

miR-19b attenuate levels of TNF-*α* suggesting that this miRNA decrease TNF-*α*-induced apoptosis of ECs [58]. In HIV-infected cardiovascular disease patients, miR-210 is positively related to the expression of TNF-*α* [97]. miR-17-3p and miR-31 target ICAM-1 and E-selectin proteins so that transfecting HUVECs with these miRNAs mimics and then stimulation with TNF decreased TNF-induced expression of these two cell surface adhesion molecules [55]. Another miRNA that inhibits ICAM-1 expression to mediate inflammation in atherosclerosis is miR-222 which is carried by endothelial microparticles [98]. Increased miR-451 expression downregulates macrophage migration inhibitory factor (MIF), a multipotent cytokine with regulatory roles in inflammatory processes, in tumor biopsies of gastric cancer patients [99, 100]. miR-100 indirectly affects cytokines so that it inhibits the mammalian target of rapamycin (mTOR) in mice with hindlimb ischemia [101]. As it was previously shown that suppression of mTOR by rapamycin reduces cardiac TNF-*α* concentration, miR-100 might exert the same decreasing effect on TNF-*α* during CAD [102]. Some CAD-related miRNAs including miR-1, miR-133, miR-146a, miR-155, miR-206, miR-208, miR-431, miR-486, miR-499, and miR-181a alter the gene expression of proinflammatory cytokines such as IL-6 and TNF-*α* during inflammation in sarcopenia [103].

## 6. Conclusions

Although a large number of studies confirmed that alteration in the levels of specific cytokines, growth factors, and miRNAs represent CAD diseases, developing new therapeutics targeting cytokines and growth factors or miRNA has not been promising since cytokines and miRNA have a complicated network of interactions in CAD. In this review of CAD-related cytokines, growth factors, and miRNAs, the interaction of cytokines such as growth factors and inflammatory cytokines on CAD-related miRNAs is elucidated, which could act as biomarkers or potential targets for therapeutics.

## Figures and Tables

**Table 1:** miRNAs, cytokines and growth factors involved in the coronary artery disease.

	Increase in level				Decrease in level		
	miRNA			Cytokine & growth factor	miRNA		Cytokine & growth factor
CAD	miR-1	miR-17-5p	miR-93	INF-*γ*	miR-10a	miR-29a/b	TGF-*β*
	miR-21	miR-133a-3p	miR-502	TNF-*α*	miR-17	miR-30e-5p	
	miR-23a	miR-146a/b	miR-486	IL-1*β*	miR-19a	miR-92a/b	
	miR-24	miR-191-3p	miR-454	IL-18	miR-22	miR-93-5p	
	miR-25	miR-361-5p	miR-765	NogoB/Nus1	miR-23a	miR-126-5p	
	miR-33a	miR-560-5p	miR-487a	IL-8	miR-31	miR-140-3p	
	miR-34a	miR-574-5p	miR-221	TL1A/R	miR-126	miR-181d	
	miR-92a	miR-615-5p	miR-222	Adamts5	miR-133	miR-199a	
	miR-122	miR-313-5b	miR-223	CRP	miR-145	miR-195	
	miR-133a	miR-370	miR-206		miR-147	miR-222	
	miR-126	miR-106b	miR-340		miR-149	miR-342	
	miR-134	miR-103a	miR-451		miR-150	miR-378	
	miR-135	miR-199a	miR-624		miR-155	miR-424	
	miR-191	miR-208a	miR-545		miR-182	miR-584	
	miR-197	miR-502	miR-215		miR-720		
	miR-2861						

CA	miR-21	miR-99a		IL-1*β*	miR-100	miR-1181	—
	miR-92a	miR-370		IL-6, IL-9	miR-8059	miR-6816-3p	
	miR-486-5p			TNF-*α*	miR-138-2-3p		

UA	miR-320a	miR-92a	miR-1	IL-1*β*	miR-19a/b		IL-2
	miR-133a	miR-186	miR-126	IL-2, IL-8	miR-126		IL-4
	miR-208b	miR-210	miR-451	IL-9, IL-10	miR-132		IL-10
	miR-135a	miR-483	miR-486a	INF-*γ*	miR-147		
	miR-140-3p	miR-499		IL-17 MCSF	miR-150		
	miR-142-5p	miR-134a-5p		MCP-1			

AMI	miR-208	miR-133a	miR-21	IL-6	miR-106		—
	miR-34a	miR-320a	miR-92a	IL-1Ra	miR-197		
	miR-423	miR-486a	miR-1	TNF-*α*	miR-223		
	miR-126	miR-126-5p	miR-133				
	miR-29b	miR-145-3p	miR-192				
	miR-194	miR-17-5p	miR-328				
	miR-499						

STEMI	miR-1			IL-2	miR-15a-5p	miR-320a	IL-6
	miR-30d			IL-6	miR-19a-5p	miR-134-5p	
	miR-133			IL-8	miR-30e-5p	miR-25-5p	
	miR-208			IL-10	miR-30a-5p	miR-16-5p	
	miR-423				miR-101-3p	miR-183-5p	
	miR-499				miR-101-5p	miR-10a	
					miR-328-5p	miR-107	
					miR-744-5p	miR-375	
					miR-103a-3p		
					miR-1307-5p		
					miR-409-5p		
					miR-181b-5p		
					miR-181a-5p		

CAD: coronary artery disease; CA: coronary atherosclerosis; UA: unstable angina; AMI: acute myocardial infarction; STEMI: ST-segment elevated myocardial infarction; Ra: receptor antagonist; MCSF: macrophage colony-stimulating factor; MCP-1: monocyte chemoattractant protein-1; INF-*γ*: interferon-gamma, TNF-*α*: tumor necrosis factor-alpha; IL: interleukin; CRP: C-reactive protein.
